# Synthesis and Application of a Novel Metal–Organic Frameworks-Based Ion-Imprinted Polymer for Effective Removal of Co(II) from Simulated Radioactive Wastewater

**DOI:** 10.3390/polym15092150

**Published:** 2023-04-30

**Authors:** Li Yu, Tu Lan, Guoyuan Yuan, Chongxiong Duan, Xiaoqin Pu, Ning Liu

**Affiliations:** 1 College of Chemistry and Chemical Engineering, Chongqing University of Science and Technology, Chongqing 401331, China; 2 Key Laboratory of Radiation Physics and Technology of the Ministry of Education, Institute of Nuclear Science and Technology, Sichuan University, Chengdu 610064, China; 3 School of Materials Science and Energy Engineering, Foshan University, Foshan 528231, China

**Keywords:** removal, Co(II), radionuclide, MOFs, ion-imprinted polymer, DFT calculations

## Abstract

In this work, a novel metal–organic frameworks (MOFs)-based ion-imprinted polymer (MIIP) was prepared to remove Co(II) from simulated radioactive wastewater. The batch experiments indicated that the sorption was well described by the pseudo-second-order kinetic and Langmuir models, and it is monolayer chemisorption. The theoretical maximum sorption capacity was estimated to be 181.5 mg∙g^−1^, which is by far the reported maximum value of Co(II) sorption by the imprinted materials. The MIIP presented an excellent selectivity for Co(II) in the presence of common monovalent and divalent metal ions, and the selectivity coefficients were 44.31, 33.19, 10.84, 27.71, 9.45, 16.25, and 7.60 to Li(I), K(I), Mg(II), Ca(II), Mn(II), Ba(II), and Cd(II), respectively. The sorption mechanism was explored by X-ray photoelectron spectroscopy (XPS) technology and density functional theory (DFT) calculations, suggesting that Co(II) was adsorbed by the MIIP via the chelation of 4-vinylpyridine (VP) ligands with Co(II), which was a spontaneous process, and the optimal coordination ratio of VP to Co(II) was 6. This work suggested that the MIIP has a high sorption capacity and excellent selectivity for Co(II), which is of great significance for the selective separation of Co-60 from radioactive wastewater.

## 1. Introduction

With the rapid development of nuclear technology, more and more radioisotopes have been produced for radiochemotherapy, scientific research, and other aspects. ^60^Co is one of the most common radioactive isotopes and is often used in irradiation breeding, non-destructive testing, and radiotherapy [1]. During the production and use of ^60^Co, a large amount of radioactive wastewater containing ^60^Co will be produced. If not treated effectively and discharged directly into the environment, it will cause serious harm to humans and ecology such as aplastic anaemia and leukaemia [2]. For this reason, many methods were adopted to remove cobalt ions from wastewater, such as extraction [3], precipitation [4], membrane separation [1], sorption [2], etc. Among them, sorption is considered to be one of the most simple and effective processes due to its high enrichment factor, low organic reagents consumption, and operational flexibility. Various sorbents including grapheme oxide [5], silica gel [6], inorganic salt [7], clay mineral [8], polymer [9], and the doped materials [10] have been prepared for the uptake of Co(II). However, several major obstacles, such as lack of active sites, low porosity, and stability, limit seriously the practical applications in Co(II) separation. Thus, improving the sorption performance including sorption capacity, selectivity, and stability is an important issue to be resolved urgently for the sorbents.

Metal–organic frameworks (MOFs) are a kind of complex polymers based on the coordination of organic linkers with metal ions/clusters [11,12]. It has the advantages of large surface area and adjustable or easily modified pore structures [13,14]. Currently, hundreds of MOFs are prepared annually for the separation of various metal ions, such as U(VI) [14], Cr(VI) [15], Hg(II) [16], etc. More importantly, some MOFs have high sorption capacities, achieving hundreds of milligrams per gram, such as the adsorption amount by Fe_3_O_4_@TMU-32 to Hg(II) [16] and ZIF-67 to U(VI) [17], all showing a great potential of MOFs in metal ion separation. We have also prepared various MOFs materials by post-synthetic modification, such as the UiO-66-Schiff base, which greatly improved the adsorption capacity of MOFs on cobalt ions. Unfortunately, MOFs were generally functionalised to improve the sorption performance by modifying the porous structures and coordination groups, which in turn weakens the selectivity of specific metal ions. Consequently, how to improve the selectivity of MOFs is the key that must be solved for potential candidates for desirable sorbents.

Ion-imprinted polymers (IIPs) are usually polymerised with metal ions and organic molecules under the action of crosslinking agents [16,18]. These two units are used as templates and functional monomers, respectively. After removing the metal ions in the imprinted materials by eluent, the obtained IIPs have good selectivity for the target metal ions. Recently, although several IIPs have been reported for the separation of Co(II) [19,20], some problems including low sorption capacity still need to be solved. In addition, preparing new IIPs with high selectivity for the separation of Co(II) is also an urgent task for the treatment of ^60^Co-containing wastewater.

In this work, a new MOF-based IIP (MIIP) was successfully prepared and used to separate of Co(II) from simulated radioactive wastewater with UiO-66-NH_2_ with good stability and high specific surface area as the matrix and 4-vinylpyridine (VP) as the ligand monomer, and it was detected based on the technology of XRD, FTIR spectroscopy, zeta potentials, N_2_ sorption–desorption measurements, and SEM. The resultant MIIP was first employed to systematically investigate the removal of Co(II) using batch sorption experiments, and a currently reported maximum sorption value of 181.5 mg∙g^−1^ for Co(II) at pH 8.3 was achieved with excellent selectivity under the experimental condition. The sorption mechanisms were also explored by the XPS and DFT methods. This work provided a novel insight into the preparation of IIPs based on MOFs and paved a new way for the tailoring of MOFs-based IIPs with high sorption performance and excellent selectivity to radionuclides.

## 2. Materials and Methods

### 2.1. Materials

Zirconium tetrachloride (ZrCl_4_), NH_2_-H_2_BDC, 3-bromopropene (BP), 4-vinylpyridine (VP), azobisisobutyronitrile (AIBN), and ethyleneglycol dimethacrylate (EGDMA) were purchased from Shanghai Macklin Technology Factory. *N*,*N*-Dimethylformamide (DMF), ethanol (EtOH), HCl, HNO_3_, NaOH, and the other metallic salts including Co(NO_3_)_2_ were obtained from Chongqing Chuandong Chemical Reagent Factory. All reagents were directly used without further purification.

### 2.2. Synthesis of MIIP

**Figure 1:**
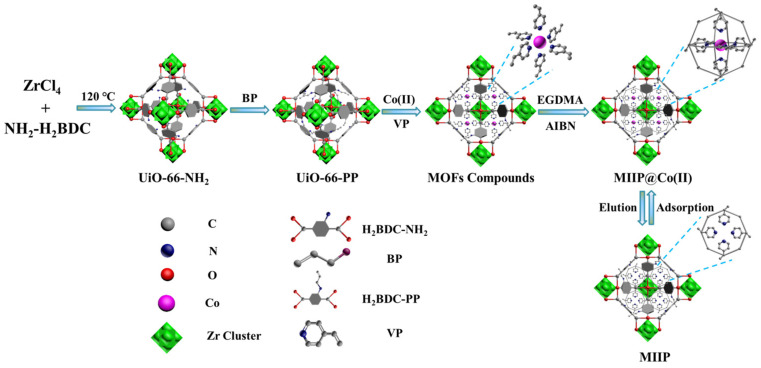
Schematic illustration for the preparation of the MIIP.

A schematic illustration to prepare the MOF-based IIP (MIIP) is shown in Figure 1. According to our previous work [21], UiO-66-NH_2_ was prepared using hydrothermal technology, followed by propylene functionalised UiO-66-NH_2_ preparation (named UiO-66-PP). Briefly, 0.5 g BP and 1.0 g UiO-66-NH_2_ were dissolved in 50.0 mL EtOH, stirring for 1.0 h, and 1.0 g K_2_CO_3_ was then added into the above solution with reflux for 3.0 h. The solid was washed with EtOH at least 3 times and dried in a 60 °C environment for 4.0 h to obtain UiO-66-PP. After that, the MIIP was prepared by surface imprinting technology. Specifically, 1.0 g UiO-66-PP, 0.6 mL VP, and 0.3 g Co(NO_3_)_2_ were added into a 250 mL flask and stirred for 2.0 h, followed by the addition of 0.1 mL EGDMA and 50 mg AIBN with crosslinking polymerisation reaction under nitrogen atmosphere for 4.0 h. The resultant particles were eluted with 1.0 mol∙L^−1^ HNO_3_ and deionised water until no cobalt ions were detected in the filtrate. The target product of the MIIP was obtained after vacuum drying at 60 °C. As a control, the MOF-based non-IIP (MNIP) was also prepared with a similar process, except that Co(NO_3_)_2_ was not added during the reaction.

### 2.3. Characterisation

The crystal structure and the functional groups of sorbents were detected based on XRD (CuKa radiation at 40 kV and 30 mA, Shimadzu, Kyoto, Japan) and FT-IR (Thermo, Waltham, MA, USA) technology. The SEM (JSM-5610LV, JEOL, Japan) was applied to measure the surface morphology of samples. The pore volume and pore size were detected based on Brunauer–Emmitt–Teller (BET) measurements (Quantachrome, MA, USA). The elemental types of sorbents before and after Co(II) sorption were analysed by XPS (XSAM800, Kratos, UK).

### 2.4. Bath Sorption Experiments

These experiments were carried out based on mixing 10.0 mg sorbent (MIIP or MNIP) with 100 mL Co(II) solution of a certain concentration in a conical flask. After adjustment of pH by NaOH or HNO_3_, the flask was placed in a rotary shaker to stir at 150 rpm at a certain temperature. After centrifugation, Co(II) concentration was detected by ICP-MS spectrometry (XSERIES 2, Thermo, Waltham, MA, USA). The sorption capacity (*q*_e_) was determined by the following equation.

(1) $$q_{e}=\frac{(C_{0}-C_{e})\times V}{m}$$

In which *C*_0_ and *C_e_* refer to Co(II) initial and equilibrium concentrations. *V* (L) represents the solution volume, while *m* (g) refers to sorbent mass.

### 2.5. DFT Calculations

To study the interaction mechanism of Co(II) and the MIIP, the DFT calculations were conducted based on the B3LYP functional with London-dispersion correction [22,23,24]. The specific DFT calculation process was presented in part S1 of the Supporting Information (SI).

## 3. Results and Discussion

### 3.1. Characterisations

**Figure 2:**
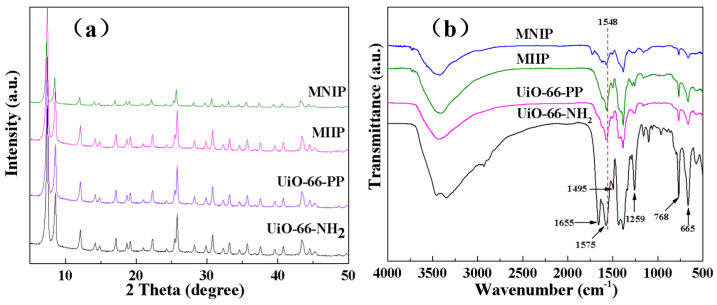
(**a**) XRD result and (**b**) FTIR spectra of UiO-66-NH_2_, MIIP, and MNIP.

The XRD results of UiO-66-NH_2_, UiO-66-PP, MIIP, and MNIP are displayed in Figure 2a. The peaks of the sample of UiO-66-NH_2_ are consistent with the simulated one, indicating its successful preparation. The XRD patterns of UiO-66-PP, MIIP, and MNIP are almost the same as UiO-66-NH_2_, suggesting unchanged crystal structures after the crosslinking reaction. FTIR spectroscopy was performed to verify the structure of the prepared MIIP and MNIP. In Figure 2b, the peaks at 1575 and 665 cm^−1^ correspond to the interaction of –COOH with Zr(IV), and the stretch vibrations at 1259 and 768 cm^−1^ are ascribed to C–N and N–H, respectively [21,25,26]. After crosslinking, a new peak at 1548 cm^−1^ associated with the stretching vibration of pyridine was observed for both the MIIP and MNIP, indicating that the crosslinking reaction was successful and the ligands were grafted onto the sample [27].

The surface area and pore structure of the MIIP and MNIP were investigated by N_2_ sorption–desorption isotherms and the results can be seen in Table 1. Clearly, the surface area, pore diameter, and volume of UiO-66-NH_2_ were estimated to be 964.2 m^2^∙g^−1^, 3.8 nm, and 0.35 cc∙g^−1^. After crosslinking, the surface area (SA) and pore volume (PV) of the MIIP (672.8 m^2^∙g^−1^ and 0.28 cc∙g^−1^) and MNIP (399.1 m^2^∙g^−1^, 0.20 cc∙g^−1^) decreased substantially, resulting from the occupied pores of UiO-66-NH_2_ by dispersed pyridines. Moreover, the porosity of MNIP is lower than MIIP due to the lack of cobalt ions template, leading to a large number of pyridines being crosslinked to MNIP.

**Figure 3:**
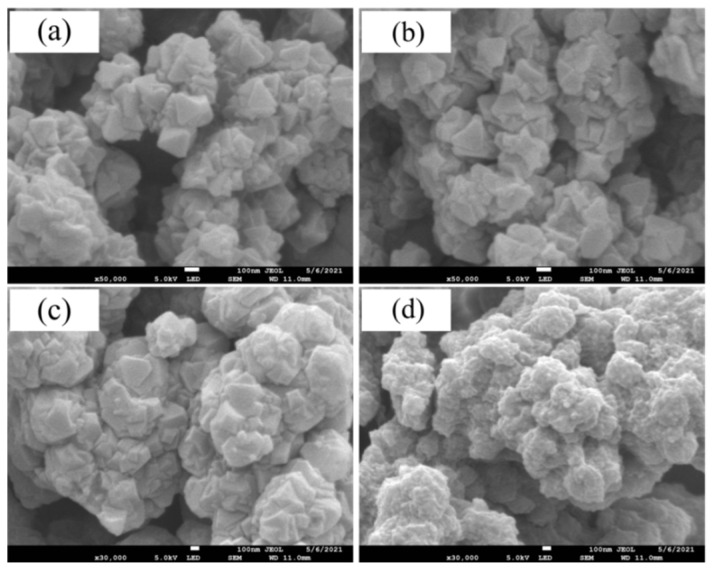
SEM images of (**a**) UiO-66-NH_2_, (**b**) UiO-66-BP, (**c**) MIIP, and (**d**) MNIP.

The morphology of UiO-66-NH_2_, UiO-66-BP, MIIP, and MNIP were characterised by SEM in Figure 3. According to the result, it can be seen that the UiO-66-NH_2_ structure in Figure 3a is a regular hexagonal structure with smooth surfaces. When grafted by BP, its surface morphology is likely to remain unchanged (Figure 3b). However, after crosslinking, the surfaces of MIIP and MNIP gradually become rough (Figure 3c,d); especially, the MNIP surface almost lacks the ribbed structure of crystals. This is mainly caused by the lack of a template agent, resulting in the abundant polymer crosslinking on the crystal surface. The composition distribution of MIIP and MNIP is presented in the EDS images (Section S3 of SI), including C, N, O, and Zr.

### 3.2. Sorption Experiments

#### 3.2.1. Effect of pH

**Figure 4:**
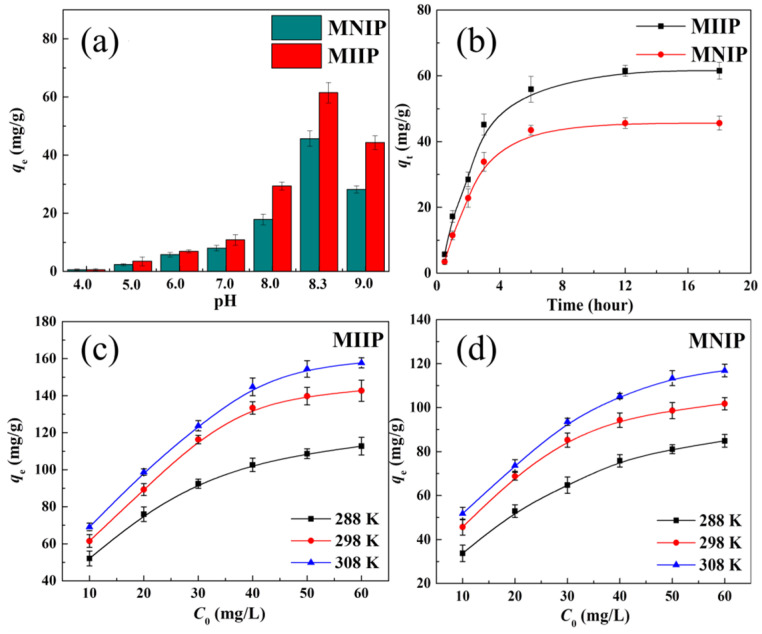
Influence of (**a**) pH, (**b**) contact time, (**c**,**d**) temperature, and initial Co(II) concentration on Co(II) sorption by MIIP and MNIP.

The sorption of Co(II) by MNIP and MIIP in the range of pH 4.0 to 9.0 is shown in Figure 4a. It is found that the sorption volume of Co(II) on both samples was enhanced significantly with increasing pH from 4.0 to 8.3 and then decreased at pH 9.0. This may be because at pH < 8.5, the cobalt ions are present in the ionic state, and the precipitation of Co(OH)_2_ would appear at pH > 8.5 [28]. In addition, the sorption sites on materials may be occupied by H^+^ in an acidic media with pH < 6.5 (Section S4 of SI), resulting in a low sorption capacity. Therefore, the optimal pH of the MNIP and MIIP for Co(II) sorption was chosen to be 8.3 in subsequent experiments. Moreover, the maximum sorption value of Co(II) by MIIP (61.5 mg∙g^−1^) is significantly larger than that by MNIP (45.6 mg∙g^−1^). This is mainly due to the more suitable pore structures of MIIP for Co(II) sorption originating from the template effect of cobalt ions. For MNIP, the lack of templates in the preparation resulted in a low sorption capacity owning to the excessive crosslinking with low porosity. These results were consistent with the analyses of SEM and BET.

#### 3.2.2. Sorption Kinetics

Figure 4b shows the influence of contact time on Co(II) sorption by MIIP and MNIP. It can be seen that the sorption amount grew rapidly in the first 3 h, then decreased to equilibrium after 12 h. The sorption kinetics were investigated by the following models, the pseudo-order kinetic model and the intra-particle diffusion model. The kinetic equations and fitted curves were shown in Section S5 of SI, while the kinetic parameters were collected in Table 2. The higher *R*^2^ values for the MNIP (0.984) and MIIP (0.990) indicated that the Co(II) sorption by MIIP and MNIP conformed to the pseudo-second-order kinetic model, which is a complexation exchange process in which the ligand groups acted as complexation exchangers [29].

#### 3.2.3. Sorption Thermodynamics

The effect of temperature on Co(II) sorption by the MIIP and MNIP was displayed in Figure 4c,d. Clearly, the sorption amount increased significantly with the increasing temperature. The thermodynamic parameters including Δ*H*^0^ (standard enthalpy change), Δ*S*^0^ (standard enthopy change), and Δ*G*^0^ (standard Gibbs free energy change) were calculated according to the sorption isotherms (Section S6 of SI), and the thermodynamic equilibrium constant (*K_d_*) was calculated based on the Khan method [30]. The resultant thermodynamic parameters are tabulated in Table 3. The Δ*H*^0^ and Δ*G*^0^ value indicated that the sorption of Co(II) by the MNIP and MIIP was spontaneous.

#### 3.2.4. Sorption Isotherms

The influence of initial Co(II) content on Co(II) sorption by the MIIP and MNIP was also studied in Figure 4c,d. Apparently, both sorption capacities increased substantially and gradually reached saturation with the increasing initial Co(II) content. The sorption volume of Co(II) by MIIP is significantly higher than that of MNIP. To evaluate the max sorption volume and obtain the sorption model of MNIP and MIIP for Co(II), the Langmuir isotherm and Freundlich isotherm models were used to fit the above sorption data, shown in Section S7 of SI and Table 4. It is found that the fitted parameters by the former model have higher correlation coefficients compared to those fitted by the later model, suggesting that the sorption of Co(II) by MIIP and MNIP are slightly more in line with the former model, which is homogeneous sorption. The maximum sorption capacity (*q*_max_) of Co(II) by MIIP can be calculated to be 181.5 mg∙g^−1^ at 308 K, which is by far the maximum value in the imprinted materials (Table 5), indicating that MOFs as a matrix for the preparation of imprinted materials have high application value for efficient removal of Co(II).

#### 3.2.5. Selectivity and Reusability

Since ^60^Co often coexists in radioactive wastewater with other metal ions, the selectivity of sorbents to separate Co(II) from a mixed metal ions solution is the key for the treatment of this kind of wastewater. Therefore, we replace Co-60 with cobalt ions and configure simulated radioactive wastewater, that is, radioactive wastewater containing various metal ions in the real environment. In order to make the interfering ions in solution closer to the actual case of radioactive wastewater, the initial concentration of each metal ion in solution is chosen to be 1.0 ppm and the solution pH is 7.5.

**Figure 5:**
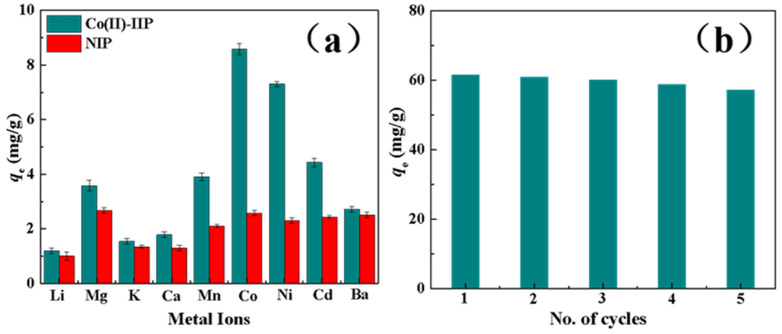
(**a**) Adsorption capacity of Co(II) onto the MNIP and MIIP with competitive metal ions presented and (**b**) reusability of the MIIP.

As illustrated in Figure 5a, the sorption amounts of the MIIP for all metal ions are higher than those of the MNIP, and the sorption efficiency of Co(II) by the MIIP is also significantly higher than those of other competitive ions. As illustrated in Table 6, the *K_d_* values of the MIIP for all ions are relatively high when compared with the MNIP. Especially for Co(II), the distribution coefficient of the MIIP is 60.42 mL∙g^−1^, which is significantly larger than other competitive ions, and its selectivity coefficient for Li(I), K(I), Mg(II), Ca(II), Mn(II), Ba(II), and Cd(II) is 44.31, 33.19, 10.84, 27.71, 9.45, 16.25, and 7.60, respectively. These results indicate that the preparation of MIIP using cobalt ions as templates and VP as ligand is successful and has an excellent selectivity for Co(II) as expected.

**Figure 6:**
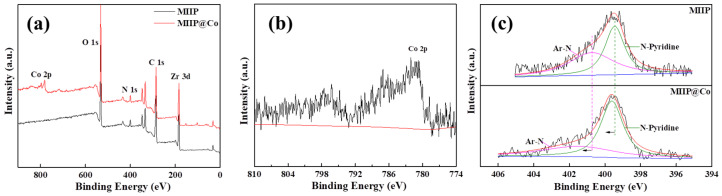
(**a**) Typical XPS survey spectra of the MIIP and MIIP@Co(II), (**b**) Co 2p, and (**c**) high resolution XPS spectra of N 1s.

The reusability of sorbents is also one of the most important parameters to evaluate potential applications in actual radioactive wastewater. This performance has a significant impact on the economy of the adsorbent, which directly determines its application value in the field of actual polluted wastewater treatment. Therefore, this paper conducts some experimental comparative studies in the research process to determine the change in the adsorption capacity of this adsorbent under the condition of a certain number of repeated adsorption cycles, providing support for its practical application. Herein, the adsorbed MIIP was eluted by HNO_3_ solution with a content of 1.0 mol∙L^−1^ and used directly for Co(II) sorption after drying. In Figure 6b, the sorption amount of the MIIP is more than 90% of the original sorption capacity even after five cycles, and XRD (Figure S8) and BET (Table S1) remain almost constant, suggesting that the prepared MIIP is a stable and efficient sorbent with a great potential for the separation of ^60^Co from radioactive wastewater.

### 3.3. Sorption Mechanism

To probe the sorption mechanism of Co(II) by the MIIP, XPS test was used to conduct the characterisation of sorbents before and after Co(II) sorption. Based on the structural characteristics of MIIP, the typical XPS survey was shown in Figure 6. Clearly, a new peak of Co 2p in Figure 6a was observed after Co(II) sorption by the MIIP (MIIP@Co), confirming that Co(II) was adsorbed on the MIIP surface. In Figure 6c, it can be seen that the N 1s spectra could be divided into two main peaks: the nitrogen on MOFs (Ar–N at 400.7 eV) and on the ligand of pyridine ring (N–Pyridine at 399.5 eV). After Co(II) sorption, the binding energies of the two peaks enhanced by 0.7 (Ar–N) and 0.2 eV (N–Pyridine), respectively, indicating that both kinds of nitrogen atoms have been complexed with cobalt ions. A previous study has shown that [34] a nitrogen atom can form coordination bonds with metal ions because of the lone pair electrons on the N atom. Therefore, the shifts from the binding energies of nitrogen atoms in Figure 6b can be supposed to be the simultaneous chelation of Co with two kinds of N atoms, which also could improve the sorption property of the sorbent.

**Figure 7:**
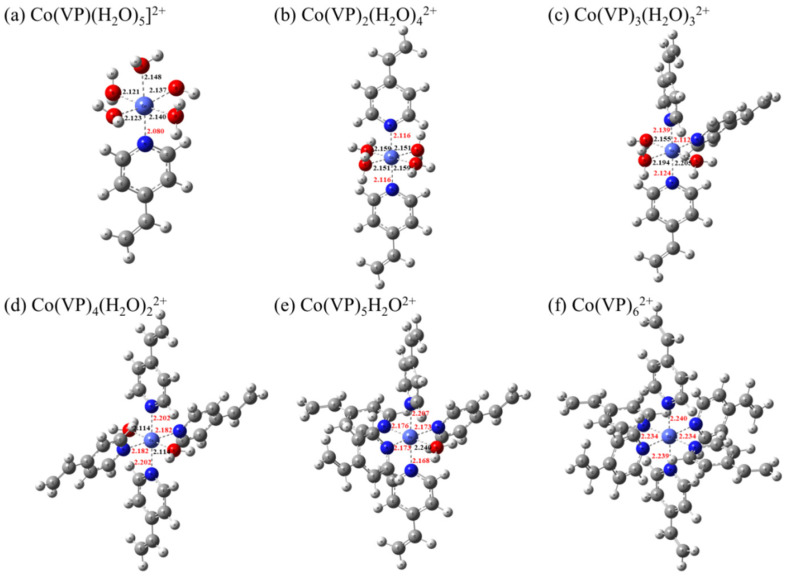
Optimised structures of Co(II)–VP complexes by DFT with the critical bond lengths (Å) in figures (Co–O_w_ bonds in black; Co–N_VP_ bonds in red).

An excellent Imprinted polymer sorbent usually requires monomers with strong complexation ability to metal ions. Therefore, to further investigate the sorption mechanism of this process, the interaction between the preselected imprinting monomer (VP) and cobalt ion was investigated using DFT calculations. In an aqueous solution, the Co(II) is surrounded by six water molecules to form Co(H_2_O)_6_^2+^, and the average bond length of water (O_W_) is about 2.115 Å [26]. Since the N atoms on the VP monomer can coordinate to Co(II), the possible structures between Co(II) and different amounts of VPs (from one to six) were optimised and the stationary points are displayed in Figure 7. It is clear that with the addition of one VP monomer, the nitrogen atom on VP can coordinate with Co to form a monodentate complex Co(VP)(H_2_O)_5_^2+^ by taking Co^2+^ from Co(H_2_O)_6_^2+^ (Figure 7a), and the bond lengths of Co–O_w_ and Co–N_VP_ are 2.134 and 2.208 Å, respectively. In Table 7, the change of Gibbs free energy with a negative value (Δ*G* = −4.85 kJ∙mol^−1^) indicated a spontaneous sorption process, and the negative sorption energy (Δ*E* = −10.95 kJ∙mol^−1^) suggests a strong affinity of VP to Co(II). As more VP monomers got involved in the coordination, both Δ*G* and Δ*E* became increasingly negative. It can be judged that the reaction speed will be significantly improved when the VP monomer increases in the reaction process, and the corresponding spontaneous tendency will be stronger, which can promote the adsorption process. The lower values indicate that the reaction is more likely to occur [35], and the most stable coordination structure, Co(VP)_6_^2+^, is presented in Figure 7f, which contains six VP ligands and a cobalt metal centre. In addition, the natural bond orbital research was conducted to investigate the Wiberg bond indices (WBIs) in Figure 7, and the result can be seen in Section S8 of SI. The average WBIs of Co–N_VP_ in complexes are in the range of 0.082–0.111, which are noticeably more than those of Co–O_W_ (0.067–0.088), which further confirmed that the nitrogen atoms of VP monomer have a strong bonding ability with Co(II), resulting in the formation of the hexadentate complex Co(VP)_6_^2+^ by six VP monomers. These results indicated that the VP molecule can be an alternative candidate as an imprinting monomer and may exhibit huge potential for the efficient elimination of cobalt ions.

## 4. Conclusions

An effective metal–organic frameworks-based MIIP was first prepared for the selective separation of Co(II). The batch sorption experiments indicated that at pH 8.3, the sorption equilibrium of Co(II) by the MIIP was reached after 12 h and the equilibrium sorption capacity was 157.7 mg∙g^−1^ at 308 K. The spontaneous sorption process can be fitted by the pseudo-second-order kinetic and Langmuir models, and the theoretical maximum sorption volume was 181.5 mg∙g^−1^ at 308 K, which is by far the maximum value in the imprinted materials. Meanwhile, the MIIP exhibited an excellent selectivity for Co(II) with a distribution coefficient of 60.42 m∙Lg^−1^ under the condition of metal ions presented, including Li^+^, K^+^, Mg^2+^, Ca^2+^, Mn^2+^, Ba^2+^, and Cd^2+^. Moreover, XPS analysis and DFT calculations revealed that the sorption mechanism of Co(II) by MIIP was the chelation of VP ligands with Co(II), which was a spontaneous process, and the optimal coordination ratio of VP to Co(II) was calculated to be 6. This work provided novel insights into the design of IIPs with MOFs as the matrix for the preparation of an MIIP with high sorption performance and excellent selectivity and radionuclides in the applications of practical wastewater treatment.

## Figures and Tables

**Table 1:** The N_2_ sorption–desorption data of UiO-66-NH_2_, MIIP, and MNIP.

Samples	Surface Area (m^2^∙g^−1^)	Pore Diameter (nm)	Pore Volume (cc∙g^−1^)
UiO-66-NH_2_	964.2	3.8	0.35
UiO-66-PP	812.6	3.7	0.32
MNIP	399.1	4.0	0.20
MIIP	672.8	3.7	0.28

**Table 2:** Kinetic parameters for Co(II) sorption by the MIIP and MNIP.

Samples	Pseudo-First-Order			Pseudo-Second-Order			Intra-Particle Diffusion		
	*q*_e,cal_	*k*_1_	*R* ^2^	*q*_e,cal_	*k*_2_	*R* ^2^	*k*_int_	*C*	*R* ^2^
MNIP	46.7	6.18 × 10^−2^	0.977	53.2	1.31 × 10^−4^	0.984	1.25	10.7	0.753
MIIP	62.5	5.98 × 10^−2^	0.981	71.9	9.48 × 10^−5^	0.990	1.69	13.8	0.795

**Table 3:** Thermodynamic parameters for the Co(II) sorption by MIIP and MNIP.

Samples	*T* (K)	ln *K*_d_	Δ*G*^0^ (kJ∙mol^−1^)	Δ*H*^0^ (kJ∙mol^−1^)	Δ*S*^0^ (J∙mol^−1^∙K^−1^)
MNIP	288	2.38	−6.0	40.2	160.4
	298	3.36	−7.6		
	308	3.46	−9.2		
MIIP	288	3.77	−8.9	20.5	102.1
	298	3.89	−9.9		
	308	4.33	−10.9		

**Table 4:** Parameters of models for Co(II) sorption by the MIIP and MNIP.

Samples	*T*	Langmuir Constants			Freundlich Constants		
		*K*_L_	*q*_max_	*R* ^2^	1/*n*	*K*_F_	*R* ^2^
MNIP	288 K	0.064	111.1	0.998	0.456	14.9	0.983
	298 K	0.114	120.2	0.999	0.360	26.6	0.972
	308 K	0.104	139.9	0.995	0.365	29.8	0.989
MIIP	288 K	0.126	130.4	0.998	0.332	32.4	0.987
	298 K	0.131	167.5	0.994	0.351	39.8	0.975
	308 K	0.150	181.5	0.994	0.324	48.5	0.989

**Table 5:** Co(II) sorption capacity by various IIP sorbents.

Sorbents	T (K)	pH	*q_e_* (mg∙g^−1^)	Ref.
Fe_3_O_4_@TiO_2_@SiO_2_-IIP	/	8.0	35.2	[31]
Co(II)-MIIP	298	8.0	74.0	[20]
Mag@silica-CIP	298	5.0	78.9	[32]
MIP	/	4.8	92.2	[33]
IIPs	/	6.0	105.0	[19]
MIIP	308	8.3	181.5	This work

**Table 6:** Selectivity coefficients of the MIIP and MNIP.

Metal Ions	MIIP		MNIP	
	*K_d_*	*k*	*K_d_*	*k*
Co(II)	60.42		3.48	
Li(I)	1.36	44.31	1.11	3.12
K(I)	1.82	33.19	1.55	2.24
Mg(II)	5.58	10.84	3.66	0.95
Ca(II)	2.18	27.71	1.48	2.34
Mn(II)	6.39	9.45	2.66	1.31
Ba(II)	3.72	16.25	3.35	1.04
Cd(II)	7.95	7.60	3.21	1.08

**Table 7:** Thermodynamic data for Co(II)–VP complexes in an aqueous solution by DFT calculations.

Path	Sorption Reaction	Δ*G*	Δ*E*
1	Co(H_2_O)_6_^2+^ + VP = Co(VP)(H_2_O)_5_^2+^ + H_2_O	−4.85	−10.95
2	Co(H_2_O)_6_^2+^ + 2 VP = Co(VP)_2_(H_2_O)_4_^2+^ + 2 H_2_O	−8.46	−19.11
3	Co(H_2_O)_6_^2+^ + 3 VP = Co(VP)_3_(H_2_O)_3_^2+^ + 3 H_2_O	−14.02	−31.96
4	Co(H_2_O)_6_^2+^ + 4 VP = Co(VP)_4_(H_2_O)_2_^2+^ + 4 H_2_O	−10.05	−35.01
5	Co(H_2_O)_6_^2+^ + 5 VP = Co(VP)_5_H_2_O^2+^ + 5 H_2_O	−23.26	−51.74
6	Co(H_2_O)_6_^2+^ + 6 VP = Co(VP)_6_^2+^ + 6 H_2_O	−23.31	−59.73

## Data Availability

The data presented in this study are available on request from the corresponding author.

## Supplementary Materials

The following supporting information can be downloaded at: https://www.mdpi.com/article/10.3390/polym15092150/s1, Figure S1: N2 sorption–desorption isotherms of UiO-66-NH_2_, MIIP, and MNIP; Figure S2: EDS spectra of MIIP and MNIP; Figure S3: Zeta potentials of MIIP and MNIP as a function of pH; Figure S4: The pseudo-first-order kinetic model fitted curves; Figure S5: The pseudo-second-order kinetic model fitted curves; Figure S6: The intra-particle diffusion model fitted curves; Figure S7: Relationship curves between lnK_d_ and 1000/T; Figure S8: XRD of MIIP after 5 cycles. Table S1. The N_2_ sorption–desorption data of UiO-66-NH_2_, MIIP, and MNIP. Table S2: Bond length (R, Å) and Wiberg bond indices (WBIs) of Co–O_w_ and Co–N_VP_ bonds in the Co(II)–VP complexes. The O_w_ represents the oxygen atoms of water and N_VP_ represents the nitrogen atoms of the ligand VP. The average value is in blue. References [36,37,38,39,40] are cited in the supplementary materials.
