# Supplementary material for: Synthesis and Application of a Novel Metal–Organic Frameworks-Based Ion-Imprinted Polymer for Effective Removal of Co(II) from Simulated Radioactive Wastewater
Source: Polymers (Basel). 2023 Apr 30;15(9):2150. doi: 10.3390/polym15092150 (PMC10180829; doi:10.3390/polym15092150)
Supplement: Supplementary file 1 [file polymers-15-02150-s001.zip › polymers-2343860-supplementary.pdf]

Supporting Information for

Synthesis and Application of a Novel MOFs-Based Ion-Imprinted Polymer for Effective Removal of Co(II) from Simulated Radioactive Wastewater

Li Yu [1], Tu Lan [2], Guoyuan Yuan [1,*], Chongxiong Duan [3], Xiaoqin Pu [1] and Ning Liu [2,*]

1 College of Chemistry and Chemical Engineering, Chongqing University of Science and Technology, Chongqing 401331, China
2 Key Laboratory of Radiation Physics and Technology of the Ministry of Education, Institute of Nuclear Science and Technology, Sichuan University, Chengdu 610064, China
3 School of Materials Science and Energy Engineering, Foshan University, Foshan 528231, China
* Correspondence: hdyyzdt@126.com (G.Y.); nliu720@scu.edu.cn (N.L.)

Table of Contents

Section S1. Details of DFT Calculations

Section S2. N_2 Sorption-Desorption Isotherms

Section S3. EDS Spectra

Section S4. Zeta Potentials

Section S5. Sorption Kinetics

Section S6. Sorption Thermodynamics

Section S7. Sorption Isotherms

Section S8. Selectivity and Reusability

Section S9. Bond Length and WBIs from DFT Calculations

Section S1. Details of DFT Calculations

Density functional theory (DFT) methods were employed to probe the interaction between Co(II) and MIIP. In this work, the hybrid B3LYP functional,[1] as implemented in Gaussian 09 package,[2] was used to perform the DFT calculations, together with Grimme's D3 correction with Becke-Johnson damping function.[3] The cobalt atom was described using Stuttgart fully relativistic effective core potentials (RECPs) and the associated optimized basis sets.[4] The small-core RECPs replaced 10 core electrons in cobalt while the corresponding valence basis set represented the remaining 17 electrons. The nonmetallic atoms including H, C, N, and O were treated using two combined basis sets: one with the 6-31+G(d) basis set for geometry optimization and frequency calculations, and another one with the larger basis set 6-311++G(d,p) to refine energies.[5] Frequency calculations were performed on all optimized stationary points to identify the nature as minima and provide thermodynamic quantities, such as energy (E) and Gibbs free energy (G). The solvent effect of water was taken into account using the polarizable continuum model (PCM).[6] The Wiberg bond indices (WBIs) were obtained using natural bond orbital (NBO) analysis.[7]

Section S2. N_2 Sorption-Desorption Isotherms

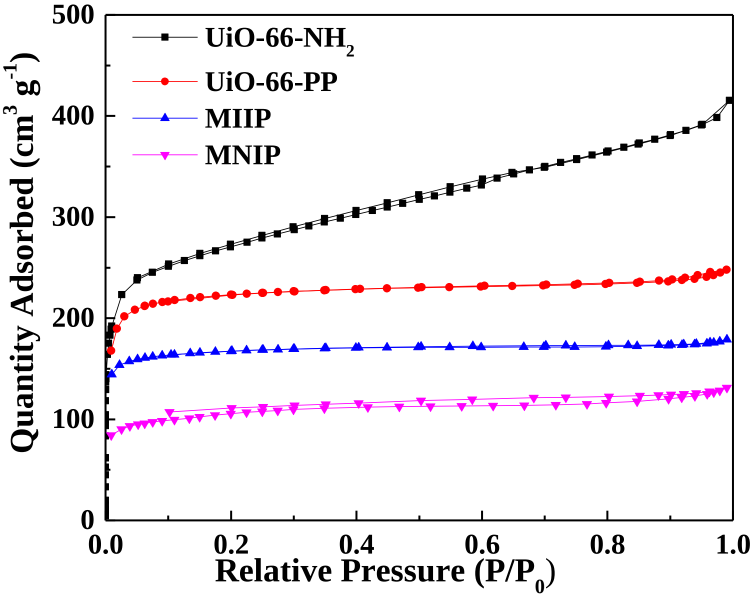

Figure S1. N_2 sorption-desorption isotherms of UiO-66-NH_2, MIIP, and MNIP.

Section S3. EDS Spectra

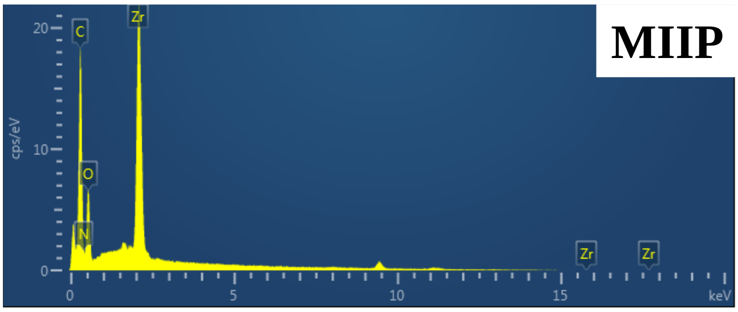

Element	Mass %	Atom %
C	45.14	62.76
N	8.83	10.52
O	21.25	22.18
Zr	24.77	4.54
Total	100.00	100.00

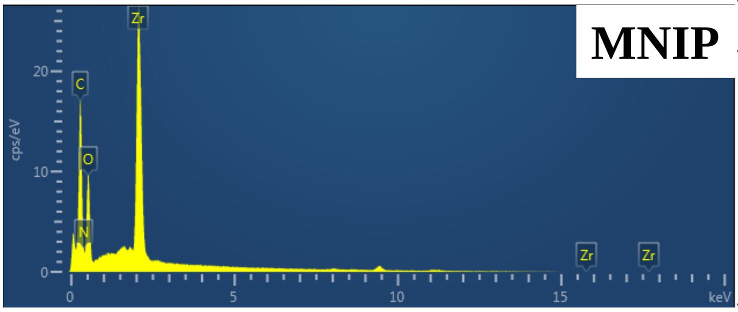

Element	Mass %	Atom %
C	50.93	68.30
N	9.01	10.37
O	17.17	17.29
Zr	22.89	4.04
Total	100.00	100.00

Figure S2. EDS spectra of MIIP and MNIP.

Section S4. Zeta Potentials

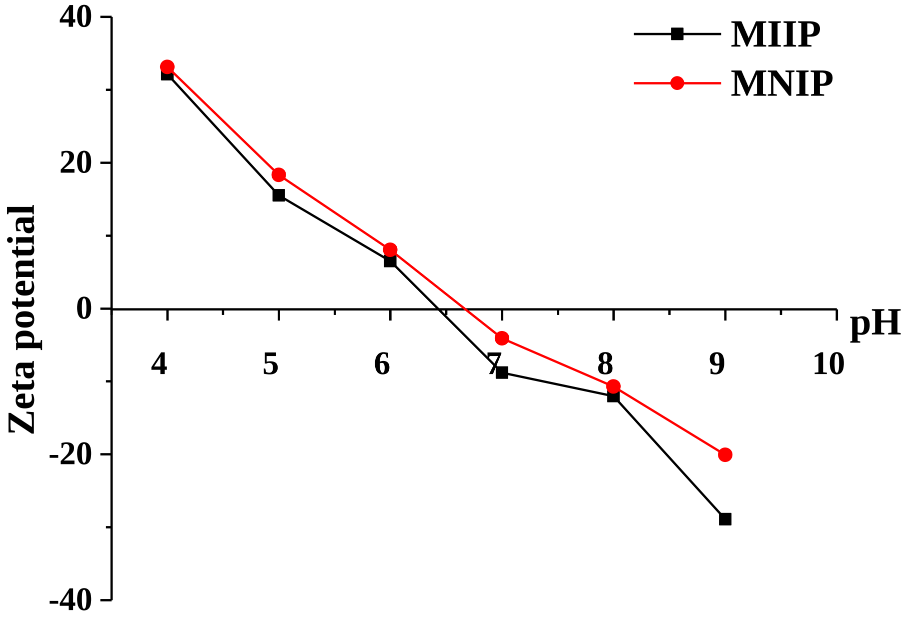

Figure S3. Zeta potentials of MIIP and MNIP as a function of pH.

Section S5. Sorption Kinetics

The pseudo-first-order (Equation S1), the pseudo-second-order (Equation S2), and the intra-particle diffusion model (Equation S3) are shown below.[8]

$$\ln(q_e - q_t) = \ln q_e - k_1 t \qquad \text{(S1)}$$

$$\frac{t}{q_t} = \frac{1}{k_2 {q_e}^2} + \frac{t}{q_e} \qquad \text{(S2)}$$

$$q_t = k_{int} t^{0.5} + C \qquad \text{(S3)}$$

where q_e (mg·g^{-1}) and q_t (mg·g^{-1}) are the amounts of metal ions adsorbed at equilibrium and at time t (min), respectively. k_1 (min^{-1}), k_2 (g·mg^{-1}·min^{-1}), and k_{int} are the kinetic rate constants for pseudo-first-order, second-order models, and intra-particle diffusion rate constants, respectively. C (mg·g^{-1}) is the constant proportional to the boundary later thickness.

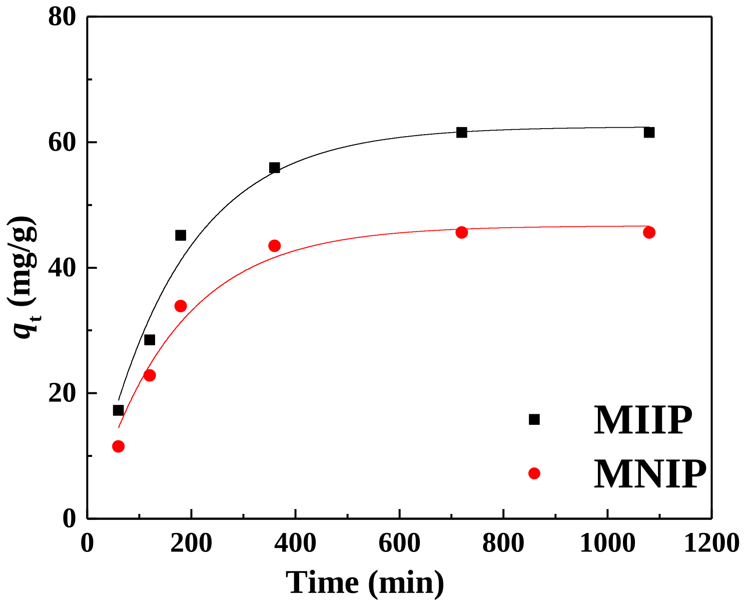

Figure S4. The pseudo-first-order kinetic model fitted curves.

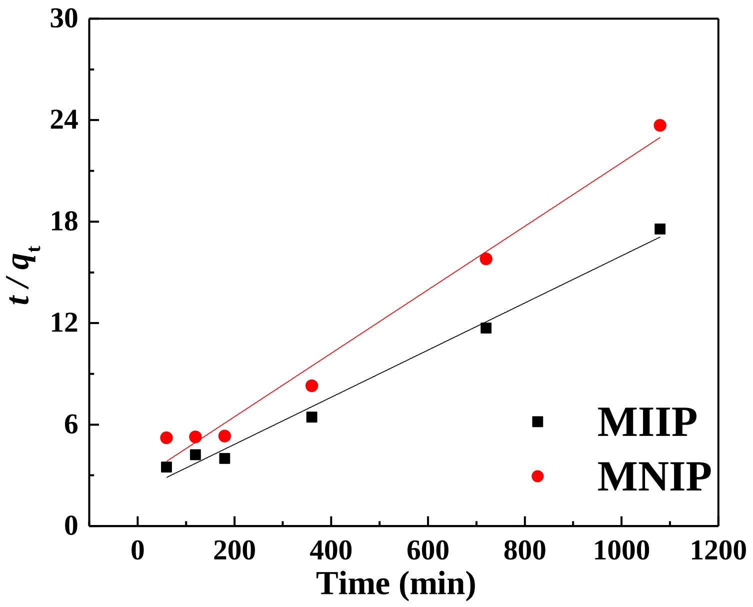

Figure S5. The pseudo-second-order kinetic model fitted curves.

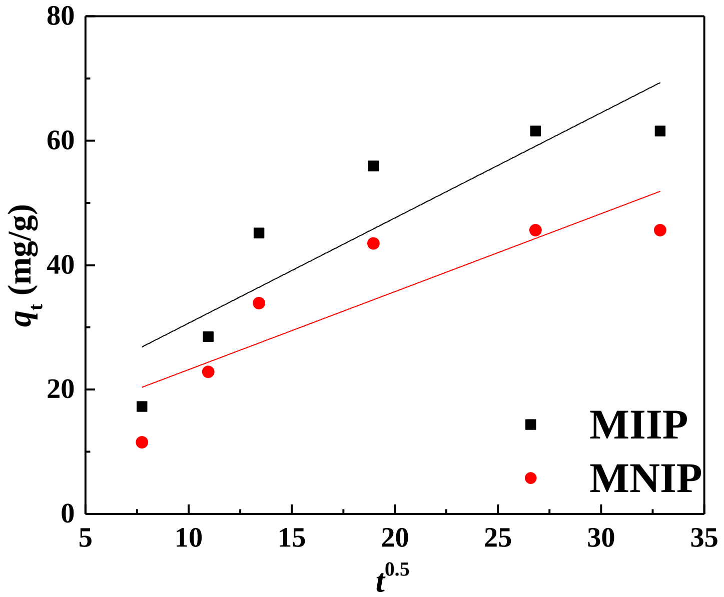

Figure S6. The intra-particle diffusion model fitted curves.

Section S6. Sorption Thermodynamics

The thermodynamic parameters including the changes of enthalpy (ΔH^0), entropy (ΔS^0), and Gibbs free energy (ΔG^0) were calculated using the following equations.

$$\ln K_d = \frac{\Delta S^0}{R} - \frac{\Delta H^0}{RT} \qquad \text{(S4)}$$

$$\Delta G^0 = \Delta H^0 - T\Delta S^0 \qquad \text{(S5)}$$

where T (K) is the absolute temperature and R (8.314 J·mol^{-1}·K^{-1}) is the ideal gas constant. The values of ΔH^0 (standard enthalpy change) and ΔS^0 (standard entropy change) were calculated from the slope and intercept of the linear regression of ln K_d *vs* 1/T.

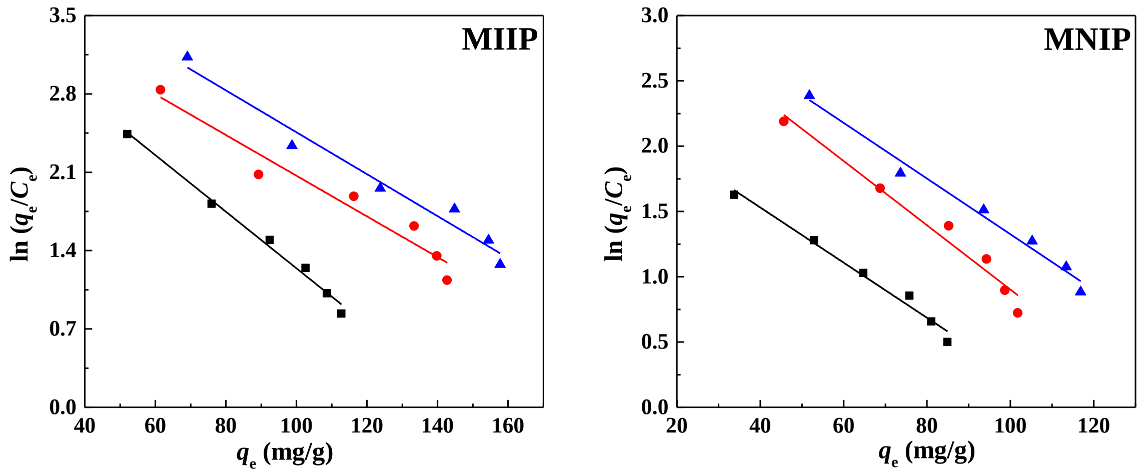

Figure S7. Relationship curves between lnK_d and 1000/T.

Section S7. Sorption Isotherms

The Langmuir model (Equation S6) and Freundlich model (Equation S7) can be described as follows.

$$\frac{c_e}{q_e} = \frac{c_e}{q_m} + \frac{1}{q_m K_L} \tag{S6}$$

$$\ln q_e = \ln K_F + \frac{1}{n} \ln c_e \tag{S7}$$

where q_m ($mg \cdot g^{-1}$) is the maximum sorption capacity, and K_L ($L \cdot mg^{-1}$) is the Langmuir sorption equilibrium constant. c_e ($mg \cdot L^{-1}$) and q_e ($mg \cdot g^{-1}$) are the concentration of the adsorbate in the equilibrated solution and the amount of the adsorbate adsorbed on the sorbent, respectively. K_F ($mg \cdot g^{-1}(L \cdot mg)^{1/n}$) is the Freundlich constant that indicates the sorption capacity, and n is an empirical parameter related to the intensity of sorption.

Section S8. Selectivity and Reusability

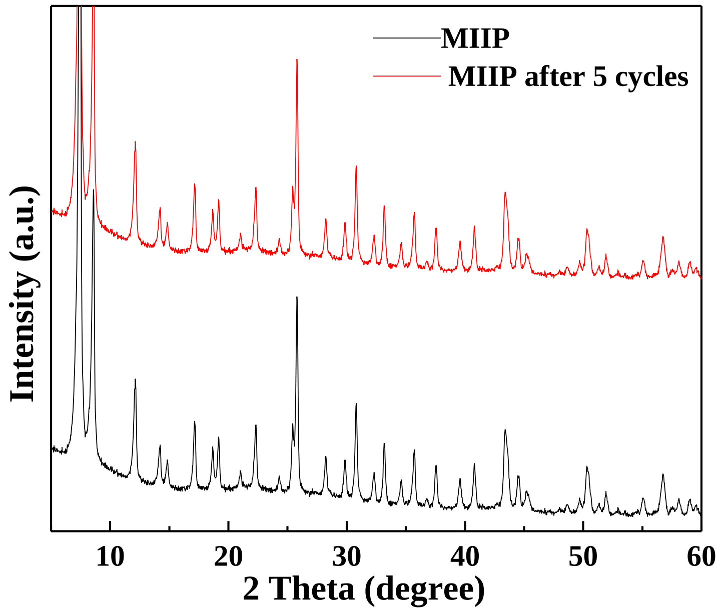

Figure S8. XRD of MIIP after 5 cycles.

Table S1. The N_2 sorption-desorption data of UiO-66-NH_2, MIIP, and MNIP.

Samples	Surface area ($m^2 \cdot g^{-1}$)	Pore diameter (nm)	Pore volume ($cc \cdot g^{-1}$)
MIIP	672.8	3.7	0.28
MIIP after 5 cycles	660.2	3.7	0.28

Section S9. Bond Length and WBIs from DFT Calculations

Table S2. Bond length (R, Å) and Wiberg bond indices (WBIs) of Co–O_w and Co–N_{VP} bonds in the Co(II)–VP complexes. The O_w represents the oxygen atoms of water and N_{VP} represents the nitrogen atoms of the ligand VP. The averaged value is in blue.

Species	Co–O_w		Co–N_{VP}	
	R (Å)	WBIs	R (Å)	WBIs
$Co(H_2O)_6^{2+}$	2.113	0.080		
	2.115	0.079		
	2.115	0.079		
	2.113	0.080		
	2.118	0.079		
	2.118	0.079		
	**2.115**	**0.079**		
$CoVP(H_2O)_5^{2+}$	2.123	0.080	2.208	0.111
	2.121	0.081		
	2.140	0.077		
	2.137	0.078		
	2.148	0.074		
	**2.134**	**0.078**		
$Co(VP)_2(H_2O)_4^{2+}$	2.159	0.076	2.116	0.103
	2.151	0.078	2.116	0.103
	2.151	0.078	**2.116**	**0.103**
	2.159	0.076		
	**2.155**	**0.077**		
$Co(VP)_3(H_2O)_3^{2+}$	2.205	0.072	2.139	0.100
	2.194	0.072	2.124	0.103
	2.155	0.080	2.112	0.108
	**2.185**	**0.075**	**2.125**	**0.104**
$Co(VP)_4(H_2O)_2^{2+}$	2.114	0.088	2.202	0.097
	2.114	0.088	2.202	0.097
	**2.114**	**0.088**	2.182	0.095
			2.182	0.095
			**2.192**	**0.096**
$Co(VP)_5H_2O^{2+}$	2.240	0.067	2.207	0.092
			2.168	0.093
			2.173	0.095
			2.173	0.094
			2.176	0.098
			**2.179**	**0.094**
$Co(VP)_6^{2+}$			2.239	0.082
			2.240	0.082
			2.234	0.082
			2.234	0.082
			2.234	0.082
			2.234	0.082
			**2.236**	**0.082**

References

(1) Becke, A. D. Density-functional thermochemistry. III. The role of exact exchange. *J. Chem. Phys.* **1993**, *98*, 5648-5652.

(2) Frisch, M. J.; Trucks, G. W.; Schlegel, H. B.; Scuseria, G. E.; Robb, M. A.; Cheeseman, J. R.; Scalmani, G.; Barone, V.; Mennucci, B.; Petersson, G. A.; Nakatsuji, H.; Caricato, M.; Li, X.; Hratchian, H. P.; Lzmaylov, A. F.; Bloino, J.; Zheng, G.; Sonnenberg, J. L.; Hada, M.; Ehara, M.; Toyota, K.; Fukuda, R.; Hasegawa, J.; Ishida, M.; Nakajima, T.; Honda, Y.; Kitao, O.; Nakai, H.; Vreven, T.; Montgomery, J. A., Jr.; Peralta, J. E.; Ogliaro, F.; Bearpark, M.; Heyd, J. J.; Brothers, E.; Kudin, K. N.; Staroverov, V. N.; Kobayashi, R.; Normand, J.; Raghavachari, K.; Rendell, A.; Burant, J. C.; Lyengar, S. S.; Tomasi, J.; Cossi, M.; Rega, N.; Millam, J. M.; Klene, M.; Knox, J. E.; Cross, J. B.; Bakken, V.; Adamo, C.; Jaramillo, J.; Gomperts, R.; Stratmann, R. E.; Yazyev, O.; Austin, A. J.; Cammi, R.; Pomelli, C.; Ochterski, J. W.; Martin, R. L.; Morokuma, K.; Zakrzewski, V. G.; Voth, G. A.; Salvador, P.; Dannenberg, J. J.; Dapprich, S.; Daniels, A. D.; Farkas, O.; Foresman, J. B.; Ortiz, J. V.; Cioslowski, J.; Fox, D. J. *Gaussian 09*, Gaussian, Inc.: Wallingford, CT, 2009.

(3) Grimme, S.; Antony, J.; Ehrlich, S.; Krieg, H. A consistent and accurate ab initio parametrization of density functional dispersion correction (DFT-D) for the 94 elements H-Pu. *J. Chem. Phys.* **2010**, *132*, 154104.

(4) Dolg, M.; Wedig, U.; Stoll, H.; Preuss, H. Energy-adjusted ab initio pseudopotentials for the first row transition elements. *J. Chem. Phys.* **1987**, *86*, 866-872.

(5) Xia, M.; Yang, X.; Chai, Z.; Wang, D. Stronger hydration of Eu(III) impedes its competition against Am(III) in binding with N-donor extractants. *Inorg. Chem.* **2020**, *59*, 6267-6278.

(6) Barone, V.; Cossi, M.; Tomasi, J. Geometry optimization of molecular structures in solution by the polarizable continuum model. *J. Comput. Chem.* **1998**, *19*, 404-417.

(7) Reed, A. E.; Weinhold, F. Natural bond orbital analysis of near-Hartree-Fock water dimer. *J. Chem. Phys.* **1983**, *78*, 4066-4073.

(8) Guo, W. L.; Chen, R.; Liu, Y.; Meng, M. J.; Meng, X. G.; Hu, Z. Y.; Song, Z. L. Preparation of ion-imprinted mesoporous silica SBA-15 functionalized with triglycine for selective adsorption of Co(II). *Colloid. Surface. A* **2013**, *436*, 693-703.
